# How breast cancer therapies impact body image – real-world data from a prospective cohort study collecting patient-reported outcomes

**DOI:** 10.1186/s12885-023-11172-y

**Published:** 2023-07-28

**Authors:** Melissa Afshar-Bakshloo, Sarah Albers, Chiara Richter, Ottilia Berninger, Jens-Uwe Blohmer, Robert Roehle, Dorothee Speiser, Maria Margarete Karsten

**Affiliations:** 1 Department of Gynecology With Breast Center, Charité – Universitätsmedizin Berlin, corporate member of Freie Universität Berlin and Humboldt-Universität Zu Berlin, Charitéplatz 1, 10117 Berlin, Germany; 2 Institute of Biometry and Clinical Epidemiology, Charité – Universitätsmedizin Berlin, corporate member of Freie Universität Berlin and Humboldt-Universität Zu Berlin, Charitéplatz 1, 10117 Berlin, Germany; 3 Berlin Institute of Health at Charité – Universitätsmedizin Berlin, Charitéplatz 1, 10117 Berlin, Germany

**Keywords:** Body image, Real-world data, Breast cancer, Patient-reported outcomes, Breast-conserving surgery, Mastectomy, Immediate breast reconstruction, Chemotherapy, Radiotherapy, Endocrine therapy

## Abstract

**Background:**

In breast cancer patients body image (BI) is a crucial aspect of quality of life (QoL). This study examined the postoperative impact of different surgical approaches on long-term BI analyzing real-world data to guide pre- and postoperative patient care and preserve QoL.

**Methods:**

EORTC QLQ-BR23 BI scores were collected electronically in 325 breast cancer patients within routine clinical care for a duration of 41.5 months (11/17/2016 – 4/30/2020) at predefined time points preoperatively and repeatedly up to two years after breast-conserving surgery (BCS) (*n* = 212), mastectomy alone (M) (*n* = 27) or mastectomy with immediate breast reconstruction (MIBR) (*n* = 86). Higher scores indicated better BI. A linear mixed regression model was used to analyze the impact of BCS, M and MIBR, as well as non-surgical therapies on BI at treatment initiation and over time.

**Results:**

BI scores deteriorated by 5 points (95%-confidence interval (CI) -8.94 to -1.57, *p*≈0.005) immediately after BCS, by 7 points (95%-CI -12.13 to -1.80, *p*≈0.008) after MIBR and by 19 points (95%-CI -27.34 to -10.34, *p* < 0.001) after M. The change over time after BCS (+ 0.10 points per week, 95%-CI -0.17 to 0.38), MIBR (-0.07 points per week, 95%-CI -0.35 to 0.20) and M (+ 0.14 points per week, 95%-CI -0.19 to 0.48) were not statistically significant (each *p* > 0.05). At treatment initiation chemotherapy was associated with a 22-point decline (95%-CI -25.39 to -17.87, *p* < 0.001) in BI score, while radiotherapy was associated with a 5-point increase (95%-CI 1.74 to 9.02, *p*≈0.004). However, over time chemotherapy was associated with a score recovery (+ 0.28 points per week, 95%-CI 0.19 to 0.37, *p* < 0.001), whereas for radiotherapy a trend towards BI deterioration was observed (-0.11 points per week, 95%-CI -0.23 to 0.02, *p*≈0.101).

**Conclusions:**

Breast cancer surgery negatively affects BI. BCS and MIBR presumably harm BI less than M in the early postoperative period. Our data suggests BI to be deteriorating in the long term after MIBR while improving after BCS or M. Radiotherapy seems to have an additional negative long-term impact on BI. These findings should be confirmed in further studies to enable evidence-based patient information as part of preoperative shared decision-making and postoperative patient care.

**Supplementary Information:**

The online version contains supplementary material available at 10.1186/s12885-023-11172-y.

## Background

Breast cancer is the most common cancer in women worldwide with over 2 million women newly diagnosed each year [1]. Over the last decades survival rates in high-income countries such as USA and Germany have improved up to a relative five-year survival rate of 90% [2, 3] which is attributed to early diagnosis due to screening programs and improved treatment [4–6]. For locoregional breast cancer tumor resection surgery is indicated [7]. Breast-conserving therapy (BCT), consisting of breast-conserving surgery (BCS) followed by adjuvant radiotherapy and mastectomy have been shown to be equivalent breast cancer treatment options regarding oncological safety [8–10]. In Western European countries 60 to 80% of newly diagnosed breast cancers can be treated with BCT [7]. However, mastectomy is the method of choice for inflammatory breast cancer, multicentric tumors, in cases of unfavorable tumor-to-breast volume ratio, incomplete tumor resection after BCS or when adjuvant radiotherapy is contraindicated [7, 11]. Except for inflammatory breast cancer, and with some caveat when combined with adjuvant radiotherapy, breast reconstruction should be considered in these cases [7, 12], administered immediately after mastectomy (immediate reconstruction) or later (delayed reconstruction) in an either implant-based or autologous fashion [13, 14]. In the process of treatment planning patients’ preferences must be considered besides other individual factors such as tumor size, localization and characteristics as well as patients’ physical constitution and state of health [11, 13, 14]. Studies have pointed out that shared decision-making, when choosing a surgical strategy improves postoperative satisfaction, body image and mental well-being in breast cancer patients [15, 16]. “Body image” is considered a multidimensional construct defined as the mental perception of one’s own body with regard to appearance, state of health, physical and social functioning as well as sexuality [17–19]. In patients with breast cancer, body image is considered a crucial aspect of quality of life [20]. Due to their disease and its therapy, cancer patients face severe changes in physical aspect and functioning [19]. According to a cognitive-behavioral model described by White, personality traits and former experiences determine, how objective and subjectively perceived bodily changes impact cancer patients’ thoughts, feelings and behavior [19]. In a patient-centered approach Hopwood et al. described three main components for the assessment of body image in cancer patients named “affective” (feeling feminine/attractive), “behavioral” (difficulties looking at oneself naked or avoiding people because of one’s appearance) and “cognitive” (satisfaction with appearance or scar) [21]. Based on this approach the widely used European Organization for Research and Treatment of Cancer (EORTC) QLQ-BR23 scale “body image” was developed [20, 21].

An impaired body image in breast cancer patients is associated with worse mental health (anxiety, depression) [15, 22–24], impaired sexuality [25, 26], as well as overall quality of life [23, 27]. Therefore, expected body image outcomes must be considered at the time of treatment decision and as part of postoperative patient care. Former studies suggest that mastectomy is more harmful to body image than BCS [23–25, 28–33], even when combined with breast reconstruction [28–30, 33–35]. Mastectomy, especially when combined with immediate breast reconstruction, is also associated with higher complication rates compared to BCS [36]. While breast reconstruction leaves the patient with a breast mold, it still cannot compensate for loss of sensation in many cases even with nerve-sparing techniques [37]. There is evidence that radio- [24, 38] and chemotherapy [25] as well as patient characteristics such as age [39–43], body-mass-index [38, 44–46], physical activity [47] and relationship status [25] have an additional influence on postoperative body image. However, long-term body image outcomes have not been sufficiently analyzed yet. Previous observations must be regarded with caution as they mainly derived from cross-sectional studies missing preoperative baseline assessments and statistical methods that control for already identified influential factors [28, 48, 49]. In this analysis prospectively collected patient-reported outcomes (PRO) were analyzed to clarify the association of different breast cancer therapies with women’s body image [50, 51] answering the following questions: (1) What impact do BCS and mastectomy without or with immediate breast reconstruction have on women’s body image at the time of surgery as well as subsequently over time? (2) What impact do radiotherapy, chemotherapy and endocrine therapy have on women’s body image at the time of treatment initiation and over time?

## Methods

### Patients

**Fig. 1:**
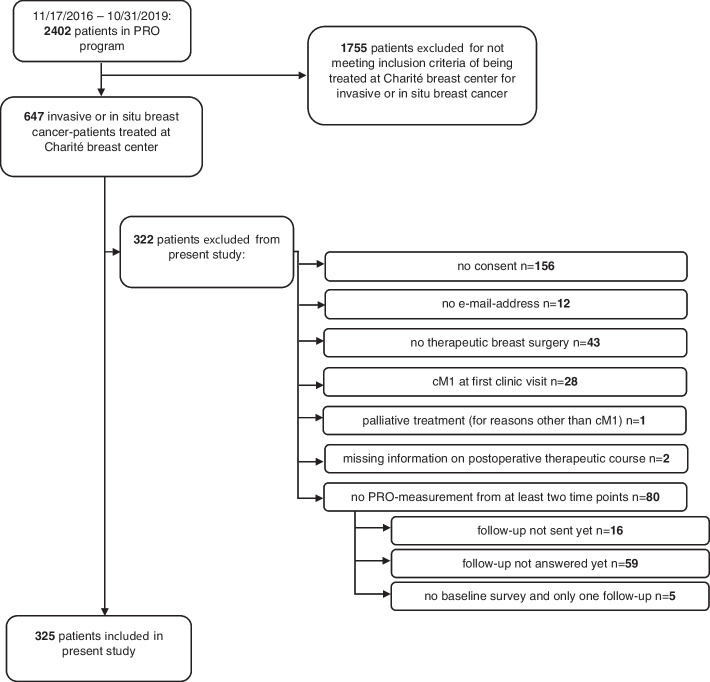
Study inclusion and exclusion criteria

Between 11/17/2016 and 04/30/2020 PROs were collected prospectively from breast cancer patients as part of an institutional ethics committee-approved (EA 4/127/16 08/30/2016) PRO program incorporated in routine clinical care at Charité – Universitätsmedizin Berlin breast center [50, 51]. Between 11/17/2016 and 10/31/2019 2402 female patients were included in the PRO program. Of those patients, 647 (647/2402, 26.9%) were treated at Charité breast center for invasive breast cancer or ductal carcinoma in situ (DCIS) and therefore eligible for follow-up survey. Eventually 325 (325/647, 50.2%) patients were included in the present study [50, 51] for meeting the following criteria: They had their first clinic visit at Charité breast center between 11/17/2016 and 10/31/2019, had given written consent for PRO collection and had received BCS, mastectomy without or with immediate breast reconstruction for invasive breast cancer or DCIS. Exclusion criteria are illustrated in Fig. 1. The observation period for the present study ended 04/30/2020 assuring that all patients were clinically monitored for a minimum of six months. Within this time frame the diagnostic process was expected to be completed allowing for treatment initiation and the start of a follow-up-PRO-survey. The observation period could therefore consist of a maximum of 41.5 months [50] (median follow-up-time: 370 days, 25%-, 75%-quartile: 204 days, 730 days).

### PRO data collection

PRO data were collected electronically preoperatively (“baseline”) as well as 0.5 (only after BCS), 1.5, 3, 6, 9, 12, 18 and 24 months after breast surgery (“follow-up”) using a web-based software [50–52]. The core system was only available within the Charité network while an additional patient portal was hosted in a different environment to allow for access to the Web and completion of questionnaires from home via a secure connection [52]. At first clinic visit patients signed a consent form after receiving verbal and written information on the PRO program. The postoperative follow-up-PRO-surveys were sent to patients via e-mail containing an access link. In case of an unanswered follow-up-PRO-survey up to three e-mail reminders were sent every two days. In accordance with the International Consortium for Health Outcomes Measurement (ICHOM) standard set of value-based patient-centered outcomes for breast cancer, the survey comprised validated PRO measures such as EORTC QLQ-C30 and QLQ-BR23 as well as sociodemographic and medical data [53].

### EORTC QLQ-BR23

The present study analyzed the EORTC QLQ-BR23 scale “body image” [50, 51] which contains four questions (feeling physically less attractive, feeling less feminine, difficulties looking at self naked, dissatisfaction with body) referring at the past week with four response options ranging from “not at all” to “a lot” [20, 54]. The score ranges from 0 to 100 where higher scores represent better body image [20]. For interpretation of change in scores regarding clinical relevance a classification by Osoba et al. [55] is commonly applied: A change of 5 to 10 points is considered small, a change of 10 to 20 points is considered moderate and a change over 20 points is considered large [55]. The breast cancer-specific QLQ-BR23 module was developed by the EORTC as an addition to the QLQ-C30 core questionnaire to measure quality of life in cancer patients [20, 54]. Overall, the QLQ-BR23 encompasses 23 questions forming two functional scales “body image” and “sexuality” as well as three symptom scales regarding “arm symptoms”, “breast symptoms” and “systemic therapy side effects” [20]. High reliability, validity and responsivity of the widely used PRO measure were confirmed by comprehensive psychometric testing [20].

### Medical data

Clinical and tumor characteristics as well as the course of treatment were documented in the web-based installation according to the patients’ medical chart in the electronic hospital information system. Treatment for invasive breast cancer or DCIS at Charité breast center followed the recommendations of the corresponding German S3-guideline [12]. For the statistical analysis patients were grouped as BCS, mastectomy alone (M) and mastectomy with immediate breast reconstruction (MIBR) [50, 51] according to their initial type of breast surgery. It was documented if patients received a “more invasive re-operation” at some point during the observation period for reasons such as R1-resections after BCS that led to M or MIBR, loss of implant after breast reconstruction caused by postoperative complications or follow-up resection, or tumor recurrence requiring another intervention. MIBR was considered more invasive than BCS and M even more invasive than MIBR regarding the extent of change in the patient’s body and looks caused by the respective surgery. For 13/325 (4.0%) cases, in which patients underwent a more invasive re-operation before answering their first postoperative follow-up-PRO-survey, group allocation was based on type of re-operation. Regarding tumor characteristics histological type, tumor grading, estrogen- and progesterone receptor, Human Epidermal Growth Factor Receptor 2 (HER2) status, tumor size (pT) and lymph node involvement (pN) were documented.

### Statistical analysis

**Fig. 2:**
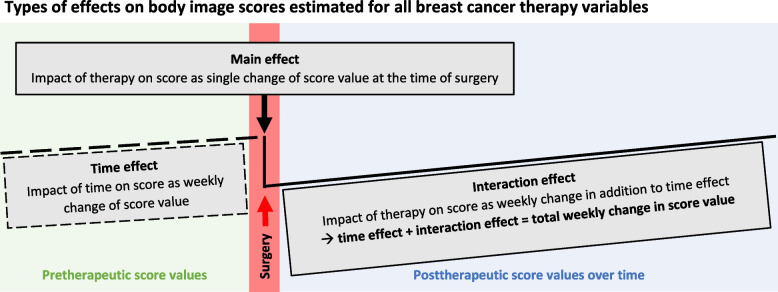
Guidance for interpretation of linear mixed regression model (after Afshar-Bakshloo et al. 2022 [51])

For statistical analysis all data were pseudonymized. Patient characteristics of the three study groups were described as appropriate either by mean or median or by absolute and relative frequency. To test the study groups for significant differences between patient characteristics apt statistical tests were performed using IBM SPSS Statistics version 26 for Microsoft Windows [56]. Scores were calculated and missing values managed according to the EORTC scoring manual which included a linear transformation of the raw score into an “S-Score” ranging from 0 to 100 points [20, 57]. A linear mixed regression model was performed with the statistical software “R” [58] including the package lme4 [59] to estimate the effect of BCS, M and MIBR as well as chemotherapy, radiotherapy and endocrine therapy and time and certain variable-time-interactions on body image scores [50, 51]. A random intercept and random slope (random slope for time) model allowed for mean differences in scores at the time of (reference) breast surgery (“intercept”) as well as varying increase (“slope”) in scores over time depending on type of therapy received. Within the designated modeling patients were under the influence of the respective therapy from the day surgery was performed or (neo)adjuvant therapy was initiated for the rest of the observation period without predefined endpoint. To adjust for other relevant predictors, selected clinical and sociodemographic characteristics were included in the analysis as covariables if clinical experience or former studies had suggested their association with body image. All models were defined before the analysis. Within the mixed model estimates with corresponding 95%-confidence interval (CI) were calculated for each independent variable regarding its effect on body image scores. Operative and (neo)adjuvant cancer therapies were time-dependent variables, while the remaining covariables (age, body-mass-index, relationship status, secondary breast cancer, lymph node involvement) were not time dependent. Figure 2 explains how estimates for time-dependent variables are interpreted. The”main effect” describes the change in score value at time point “zero”, i.e. the moment the (reference) breast surgery is performed. The “interaction effect” indicates the score development over time as a weekly change in score value from the moment the respective variable applied. Additionally, a “time effect” as a theoretical value indicates the weekly change in score values if (theoretically) none of the cancer therapy variables applied and the score was influenced only by time. For all covariables that were not time dependent the associated change in score value independently of a certain time point was estimated. R^2^ (including 95%-CI) was determined to illustrate the proportion of variance in body image scores that was explained by the mixed model. For each independent variable a partial R^2^ indicates its level of influence on the score. For the present analysis p-values were regarded explorative. As cutoff for interpretation 0.05 was chosen. For the explorative character of the present analysis, it was not adjusted for multiple testing. As the PRO program was offered to all patients at first clinic visit as part of routine clinical care, a power calculation for this analysis was not realized.

## Results

### Patient characteristics

A total of 325 out of 647 (50.2%) women with invasive breast cancer or DCIS were included in the study. Baseline characteristics were comparable between study groups except for age (mean 54.90 ± 11.09 (BCS) vs. 65.19 ± 12.01 (M) vs. 47.05 ± 10.90 (MIBR)) and menopausal status (postmenopausal: 59.3% (BCS) vs. 73.9% (M) vs. 38.0% (MIBR)) (see Table 1). Regarding tumor characteristics study groups differed in frequency of secondary breast cancer (8.5% (BCS) vs. 40.7% (M) vs. 17.4% (MIBR)), estrogen receptor positive tumors (82.6% (BCS) vs. 82.6% (M) vs. 63.7% (MIBR)), tumor grading (e.g. G3: 21.7% (BCS) vs. 19.0% (M) vs. 41.9% (MIBR)), tumor size (e.g. (y)pT1: 45.8% (BCS) vs. 14.8% (M) vs. 39.3% (MIBR) and (y)pT3: 4.2% (BCS) vs. 25.9% (M) vs. 6.0% (MIBR)) and nodal positivity (≥ (y)pN1: 16.5% (BCS) vs. 40.7% (M) vs. 25.0% (MIBR)) (see Additional file 1). Postoperative residual tumors had remained most frequently after BCS (R1: 23.0% (BCS) vs. 0.0% (M) vs. 14.8% (MIBR)), but re-operations were most common after MIBR (28.8% (BCS) vs. 11.1% (M) vs. 38.4% (MIBR)) (see Table 2). Study groups also showed differences regarding the type of axillary surgeries received, with sentinel lymph node biopsy (SLNB) predominantly performed in the BCS (71.7%) and MIBR (64.0%) groups, but in less of a third of M patients (29.6%). At the same time, M patients underwent the more invasive axillary lymph node dissection (ALND) more frequently (51.9%) than BCS (11.3%) or MIBR patients (20.9%) (see Table 2). Adjuvant radiotherapy was most common in the BCS group (82.5% (BCS) vs. 48.1% (M) vs. 23.3% (MIBR)) and chemotherapy most common in the MIBR group (36.8% (BCS) vs. 40.7% (M) vs. 62.8% (MIBR)) (see Table 2). Response rate was comparable between study groups (see Additional file 2). During the first postoperative year response rate ranged mainly between 60 to 70%, while in the second year it declined to around 25% given the fact that the minimum observation period was six months and only part of the study cohort was followed up for two years. As expected, due to predefined follow-up-scheme two weeks postoperative response rate was significantly higher in the BCS group compared to M and MIBR (ca. 50% vs. < 20% each; *p* < 0.001).

**Table 1:** Patient characteristics

	**BCS** (*n* = 212)		**M** (*n* = 27)		**MIBR** (*n* = 86)		*p*-value*
Age n (%)**	212 (100%)		27 (100%)		86 (100%)		
Mean (SD)	54.90^a^ (11.09)		65.19^b^ (12.01)		47.05^c^ (10.90)		< 0.001^ V^
**Body-mass-index (kg/m**^**2**^) n (%)**	199 (93.9%)		23 (85.2%)		79 (91.9%)		≈0.118^ K^
Median (25%-, 75%-quartile)	24.24 (21.22, 27.14)		25.47 (21.64, 27.78)		22.86 (20.66, 25.91)		
**Menopause** n (%)**	199	(93.9%)	23	(85.2%)	79	(91.9%)	≈0.001
Postmenopausal	118^a^	(59.3%)	17^a^	(73.9%)	30^b^	(38.0%)	
Premenopausal	81^a^	(40.7%)	6^a^	(26.1%)	49^b^	(62.0%)	
**Comorbidities** n (%)**	199	(93.9%)	23	(85.2%)	79	(91.9%)	≈0.551
No	106	(53.3%)	10	(43.5%)	38	(48.1%)	
Yes	93	(46.7%)	13	(56.5%)	41	(51.9%)	
**Smoking** n (%)**	199	(93.9%)	23	(85.2%)	79	(91.9%)	≈0.237^F^
Non-smoker	151	(75.9%)	17	(73.9%)	59	(74.7%)	
Active smoker	30	(15.1%)	2	(8.7%)	7	(8.9%)	
Former smoker	18	(9.0%)	4	(17.4%)	13	(16.5%)	
**Alcohol consumption** n (%)**	199	(93.9%)	23	(85.2%)	79	(91.9%)	≈0.916^F^
None	52	(26.1%)	6	(26.1%)	21	(26.6%)	
Occasional	112	(56.3%)	14	(60.9%)	48	(60.8%)	
Weekly	27	(13.6%)	2	(8.7%)	9	(11.4%)	
Daily	8	(4.0%)	1	(4.3%)	1	(1.3%)	
**Relationship** n (%)**	176	(83.0%)	21	(77.8%)	70	(81.4%)	≈0.731
None	23	(13.1%)	2	(9.5%)	9	(12.9%)	
Married/ cohabiting union	131	(74.4%)	16	(76.2%)	56	(80.0%)	
Divorced/seperated	14	(8.0%)	1	(4.8%)	4	(5.7%)	
Widowed	8	(4.5%)	2	(9.5%)	1	(1.4%)	
**Education** n (%)**	196	(92.5%)	23	(85.2%)	79	(91.9%)	≈0.624^F^
No degree	3	(1.5%)	1	(4.3%)	0	(0.0%)	
Low	10	(5.1%)	1	(4.3%)	3	(3.8%)	
Medium	45	(23.0%)	6	(26.1%)	15	(19.0%)	
High	138	(70.4%)	15	(65.2%)	61	(77.2%)	

*SD* Standard deviation

^*^Chi^2^-test was performed to test for group differences. Exceptions are indicated. In case of a p value below 0.05, a z-test for column proportions was performed as post-hoc-test

^V^Univariate ANOVA was performed to test for group differences. In case of a p value below 0.05, a Tukey-test was performed as post-hoc-test

^F^Fisher’s exact test was performed to test for group differences

^K^Kruskal–wallis-test was performed to test for group differences

^a,^^b,c^Same letters indicate that the post-hoc test for two study groups (i.e. their values) had a p value above 0.05

^**^Number of patients per group with available data. Available data < full group size indicates missing data

**Table 2:** Course of treatment

	**BCS** (*n* = 212)		**M** (*n* = 27)		**MIBR** (*n* = 86)		*p*-value*
**Residual tumor◊** n (%)**	209	(98.6%)	27	(100%)	81	(94.2%)	≈0.009
R0 (no residual tumor)	161^a^	(77.0%)	27^b^	(100%)	69^a^	(85.2%)	
R1 (microscopic residual tumor)	48^a^	(23.0%)	0^b^	(0.0%)	12^a^	(14.8%)	
**Re-operation•** n (%)**	212	(100%)	27	(100%)	86	(100%)	≈0.022
Yes	61^a^^, b^	(28.8%)	3^b^	(11.1%)	33^a^	(38.4%)	
No	151^a^^, b^	(71.2%)	24^b^	(88.9%)	53^a^	(61.6%)	
**More invasive re-operation• **n (%)**	212	(100%)	27	(100%)	86	(100%)	≈0.289
Yes	15	(7.1%)	0	(0.0%)	4	(4.7%)	
No	197	(92.9%)	27	(100%)	82	(95.3%)	
**Axillary surgery** n (%)**	212	(100%)	27	(100%)	86	(100%)	< 0.001^F^
None	34^a^	(16.0%)	4^b^	(14.8%)	12^a^	(14.0%)	
SLNB	152^a^	(71.7%)	8^b^	(29.6%)	55^a^	(64.0%)	
Axillary sample	2^a^	(0.9%)	1^b^	(3.7%)	1^a^	(1.2%)	
ALND	24^a^	(11.3%)	14^b^	(51.9%)	18^a^	(20.9%)	
**Radiotherapy** n (%)**	212	(100%)	27	(100%)	86	(100%)	< 0.001
Yes	175^a^	(82.5%)	13^b^	(48.1%)	20^c^	(23.3%)	
No	37^a^	(17.5%)	14^b^	(51.9%)	66^c^	(76.7%)	
**Chemotherapy** n (%)**	212	(100%)	27	(100%)	86	(100%)	< 0.001
Yes	78^a^	(36.8%)	11^a^	(40.7%)	54^b^	(62.8%)	
No	134^a^	(63.2%)	16^a^	(59.3%)	32^b^	(37.2%)	
**Targeted therapy** n (%)**	212	(100%)	27	(100%)	86	(100%)	≈0.087
Yes	22	(10.4%)	3	(11.1%)	17	(19.8%)	
No	190	(89.6%)	24	(88.9%)	69	(80.2%)	
**Endocrine therapy** n (%)**	212	(100%)	27	(100%)	86	(100%)	≈0.853
Yes	127	(59.9%)	15	(55.6%)	53	(61.6%)	
No	85	(40.1%)	12	(44.4%)	33	(38.4%)	

*SLNB* Sentinel lymph node biopsy, *ALND* Axillary lymph node dissection

^*^Chi^2^-test was performed to test for group differences. Exceptions are indicated. In case of a p value below 0.05, a z-test for column proportions was performed as post-hoc-test

^F^Fisher’s exact test was performed to test for group differences. In case of a *p* value below 0.05, a Fisher’s exact test for pairwise comparison was performed as post-hoc-test

^a^^, b, c^ Same letters indicate that the post-hoc test for two study groups (i.e. their values) had a *p* value above 0.05

^**^Number of patients per group with available data. Available data < full group size indicates missing data

◊Refers to resection status after reference breast surgery (that group allocation was based on)

•“Re-operations” are all surgeries performed after the initial breast surgery.”More invasive re-operations” are only the type of re-operations that for their more invasive character did not match the allocated surgical group anymore (M more invasive than MIBR, MIBR more invasive than BCS)

### Linear mixed regression model

Table 3 shows the estimated effects on body image for all analyzed independent variables as determined by the mixed model. An overall R^2^ of 25.1 (95%-CI 22.3 to 29.6) indicates fair model fit of the included variables for the complex construct of body image. Data regarding precision of model are presented in Additional file 3.

**Table 3:** Estimated effects of breast cancer therapies on EORTC QLQ-BR23 body image score (modified after Afshar-Bakshloo et al. 2022 [51] )

	**Estimate**	**SE**	**95%-CI**		**DF**	**t-value**	***p*-value**	**R**^**2a**^	**95%-CI**	
			**Lower**	**Upper**					**Lower**	**Upper**
**Intercept**	73.7998	9.6102	54.9445	92.6551	1157	7.6794	≤ 0.0001	25.1	22.3	29.6
*Main effects (Estimate* = *a single change in score value at the time of reference breast surgery)*										
** Breast-conserving surgery**	-5.259	1.8779	-8.9435	-1.5746	1157	-2.8005	0.0052	0.4	0	1.3
** Mastectomy alone**	-18.8421	4.3314	-27.3403	-10.3439	1157	-4.3501	≤ 0.0001	1.5	0.5	3
** Mastectomy with immediate breast reconstruction**	-6.9666	2.6312	-12.1291	-1.8042	1157	-2.6477	0.0082	0.5	0	1.5
** Re-operation (more invasive)**	-13.8644	5.3756	-24.4115	-3.3173	1157	-2.5791	0.01	0.4	0	1.4
** Chemotherapy**	-21.6337	1.9169	-25.3946	-17.8728	1157	-11.286	≤ 0.0001	12.5	9.6	15.8
** Radiotherapy**	5.3783	1.8565	1.7358	9.0207	1157	2.897	0.0038	0.5	0	1.5
** Endocrine therapy**	-3.1082	1.8629	-6.7632	0.5469	1157	-1.6684	0.0955	0.2	0	0.9
*Time effect (Estimate* = *a weekly change in score value caused by the mere influence of time)*										
** Time**	0.0017	0.1292	-0.2519	0.2552	1157	0.013	0.9897	0	0	0.4
*Interaction effects (Estimate* = *a weekly change in score value caused by the influence of the respective therapy, plus time effect)*										
** Breast-conserving surgery**	0.1037	0.1407	-0.1723	0.3798	1157	0.7373	0.4611	0	0	0.5
** Mastectomy alone**	0.1444	0.1706	-0.1903	0.4792	1157	0.8464	0.3975	0.1	0	0.6
** Mastectomy with immediate breast reconstruction**	-0.0733	0.1406	-0.3491	0.2025	1157	-0.5215	0.6021	0	0	0.4
** Re-operation (more invasive)**	-0.0438	0.0994	-0.2388	0.1512	1157	-0.441	0.6593	0	0	0.4
** Chemotherapy**	0.2803	0.0467	0.1886	0.372	1157	5.9989	≤ 0.0001	3.9	2.2	6.1
** Radiotherapy**	-0.1062	0.0647	-0.2331	0.0207	1157	-1.6418	0.1009	0.3	0	1.1
** Endocrine therapy**	-0.0426	0.0518	-0.1442	0.059	1157	-0.8234	0.4105	0.1	0	0.6
*Covariables (time-independent)*										
** Age (at the time of breast surgery)**	0.2449	0.1118	0.0247	0.465	258	2.1904	0.0294	1.6	0.5	3.1
** Body-mass-index**	-0.3365	0.2773	-0.8825	0.2095	258	-1.2137	0.226	0.5	0	1.5
** Relationship status**	4.8655	2.8883	-0.8222	10.5532	258	1.6845	0.0933	1.1	0.3	2.4
** Secondary breast cancer**	-0.4728	3.6463	-7.6532	6.7076	258	-0.1297	0.8969	0	0	0.4
** pN0** (no lymph node involvement)	2.1281	4.112	-5.9691	10.2254	258	0.5176	0.6052	0.1	0	0.7
** pN + **(lymph node involvement)	1.4767	4.6855	-7.7501	10.7035	258	0.3152	0.7529	0	0	0.5

*SE* Standard error, *CI* Confidence interval, *DF* Degree of freedom

^a^R^2^ indicates % of variance in scores explained by statistical model. First table row (Intercept) indicates R^2^ of model overall, remaining rows indicate partial R^2^ of respective variables

### Estimated effect of BCS, M and MIBR on body image

Immediately at the time of surgery BCS and MIBR were associated with a single 5-point (95%-CI -8.9 to -1.6, *p*≈0.0052) and 7-point (95%-CI -12.1 to -1.8, *p*≈0.0082) decline in body image score while M was associated with a single 19-point decline (95%-CI -27.3 to -10.3, *p* ≤ 0.0001) [50, 51]. No deviations with a *p* value below 0.05 from the time effect from before the surgery were found [50, 51]. However, a trend towards score recovery over time was observed for BCS (+ 5 points per year, 95%-CI -0.2 to 0.4 per week, *p*≈0.4611) and M (+ 8 points per year, 95%-CI -0.2 to 0.5 per week, *p*≈0.3975), whereas the score had a tendency towards further deterioration over time (-4 points per year, 95%-CI -0.3 to 0.2 per week, *p*≈0.6021) after MIBR [50, 51]. A more invasive re-operation was associated with a significant single 14-point decline in body image score (95%-CI -24.4 to -3.3, *p* = 0.01) [51].

### Estimated effect of chemotherapy, radiotherapy and endocrine therapy on body image

For chemotherapy a single decline in body image score of -22 points immediately at treatment initiation was estimated (95%-CI -25.4 to -17.9, *p* ≤ 0.0001) [50, 51]. This effect was modeled time-dependent and is given here as the theoretical value if the chemotherapy had started at the time of surgery. Deviations from this at other time points were only minor. However, chemotherapy was associated with a score recovery over time with a yearly 15-point gain (95%-CI 0.2 to 0.4 per week, *p* ≤ 0.0001) [50, 51]. Radiotherapy was associated with a single 5-point increase in body image score (95%-CI 1.7 to 9.0, *p*≈0.0038) at treatment initiation (i.e. at the time of breast surgery as explained above), and a trend towards deterioration over time (-6 points per year, 95%-CI -0.2 to 0.0 per week, *p*≈0.1009) [50, 51]. Endocrine therapy did not impact body image score (-3 points at treatment initiation (see above), 95%-CI -6.8 to 0.5, *p*≈0.0955, subsequently -2 points yearly, 95%-CI -0.1 to 0.1 per week, *p*≈0.4105) [50, 51].

### Estimated effect of covariables on body image

Among the included covariables only age was associated with body image scores: Estimates implied 20 additional years of age to be associated with a 5-point higher body image score (95%-CI 0.0 to 0.5 per year, *p*≈0.0294) independently of a certain time point [51].

## Discussion

Taking into account the reviewed literature, the evolution of body image after BCS, M and MIBR over time had not been sufficiently analyzed yet. Previous studies published contradictory results hinting at a potential change in body image over the postoperative time course [27, 29, 31, 60–63]. Arora et al. found a significantly worse body image some weeks after mastectomy with or without breast reconstruction compared to BCS in a population of breast cancer patients at age 60 years or younger, while six months after surgery these differences were no longer evident [29]. However, in a German multicentric prospective study by Engel et al. including 990 breast cancer patients body image improved over the postoperative course of two years after BCS, while it did not improve after mastectomy and was significantly worse one and two years after mastectomy compared to BCS (mastectomy with breast reconstruction was not included in the analysis) [62]. Besides, a more recent study by Rosenberg et al. observed an improvement in body image scores in young breast cancer patients (≤ 40 years) from one to five years after breast cancer diagnosis not only after BCS, but after uni- or bilateral mastectomy (84% had reconstruction) as well [63].

Compared to these former studies, the high-quality longitudinal PRO-collection over 24 months including a preoperative baseline-assessment and a robust statistical analysis controlling for a variety of covariables are a main strength of the present work. This meets the need to clarify the impact of different types of breast cancer surgery and (neo)adjuvant therapy on postoperative body image in order to inform patients evidence-based about potential quality of life implications as part of preoperative shared decision-making and to specifically address body image concerns over the course of postoperative patient care.

A negative impact of breast cancer surgery on body image was identified by the present analysis while controlling for age, body-mass-index, relationship status, secondary breast cancer, lymph node involvement and more invasive re-operations as well as (neo)adjuvant chemo-, radio- and endocrine therapy. At the time of surgery BCS and MIBR were associated with a 5-point and 7-point decline in scores considered therefore a clinically relevant but after Osoba et al. [55] small deterioration in body image, while M was more harmful being associated with a moderate 19-point deterioration [50, 51]. These estimates confirmed former study results [24, 28, 30, 42, 62, 64] and met our expectations from a clinical point of view. BCS is considered the least invasive type of surgery preserving as much natural breast tissue as possible and M the most invasive type resulting in a total loss of breast. Moreover, the longitudinal PRO analysis gave new insights into the differences between M and MIBR regarding their impact on body image. While IBR mitigates the damage on body image in the early postoperative period after mastectomy (-7 vs. -19 points immediately at the time of breast surgery), the procedure might have an increasingly negative effect on body image in the long term (yearly change in scores -4 vs. + 8 points) [50, 51]. As the effects of BCS, M and MIBR on body image over time did not reach statistical significance these observations must be interpreted with caution. For the uneven sample sizes in the study groups BCS (212/325, 65.2%), M (27/325, 8.3%) and MIBR (86/325, 26.5%) results for M patients must be considered less reliable than for BCS patients. This resulted from the real-world cohort evaluated within this study and was accounted for in the best way possible by the applied mixed model. Due to the limited sample size beyond one year after the initial surgery, especially results beyond that time point should be interpreted cautiously. When Rosenberg et al. observed an improving body image over time in a group of young breast cancer patients who had received mastectomy followed by breast reconstruction in over 80% of the cases, mastectomy with and without breast reconstruction were not compared or analyzed separately thus hindering conclusions regarding body image development after breast reconstruction in particular [63]. However, our findings are consistent with results from an English multicentric study (*n* = 103) by Harcourt et al. who analyzed breast cancer patients’ body image preoperatively as well as six and 12 months postoperatively comparing M (*n* = 56), MIBR (*n* = 37) and delayed reconstruction (*n* = 10): One year after MIBR body image had deteriorated compared to the preoperative baseline level in 48.6% of patients, while this was the case for only 35.7% of patients after M [41]. Moreover, type of breast surgery was significantly associated with body image over time in a repeated-measure analysis of variance [41]. Yet, these significant interactions were not detected in post hoc analysis [41]. For the small population size, the results by Harcourt et al. must be interpreted with caution. However, these findings are further supported by a larger (*n* = 1065) Finnish prospective study by Rautalin et al. that observed a decline in body image scores over 24 months after IBR [65] along with another large Taiwanese longitudinal cohort study (*n* = 741) by Konara Mudiyanselage et al. that reported worse body image after breast reconstruction compared to M from 1.5 years postoperatively on over a total follow-up of eight years [66]. From a clinical point of view these findings could be explained by reconstruction- or rather implant-associated complications such as infections, rupturing and capsular contracture resulting in revision surgeries. This goes along with the observation that re-operations overall were most common in the MIBR group (33/86, 38.4%) in the present work. The applied mixed model controlled for the sort of re-operations that were more invasive than the initial type of breast surgery (i.e. secondary mastectomy after initial BCS or loss of implant after MIBR, 4/86, 4.7%). However, other types of re-operations like follow-up resections, surgery on the contralateral breast and resection of capsular contracture, which were the most common types of re-operation in the present MIBR group are likely to have additionally affected patients’ body image and well-being overall. The time that had passed after the initial surgery and a second surgery varied between one day and over a year and some patients even experienced more than one re-operation, thus it’s possible these surgeries influenced patients’ body image over the course of two years after initial surgery. Additionally, personality traits leading to a certain therapeutic decision where clinically possible e. g. patients choosing one surgical method over the other must be considered: Patients attaching more importance to an unimpaired physical appearance might have preferred MIBR over M, while patients who were less concerned with their appearance might have chosen M more often. In the presented real-world cohort M was the least common surgery and besides for personal preference, patients probably underwent M when they were not eligible for other surgical options.

The fact that former studies by Han et al. [30] and Spatuzzi et al. [33] failed to detect these trends spotting no difference between body image scores after M with and without breast reconstruction might be explained by the lack of baseline-data in their retrospective analysis and a smaller study population (*n* = 112 and *n* = 157). Additionally, these studies did not analyze the influence of more invasive re-operations on body image, unlike the present work, which was able to detect a negative impact. This observation matches clinical expectations that re-operations pose a considerable emotional challenge for patients with associated consequences on body image. The estimated deterioration in body image scores caused by BCS or MIBR (-5 and -7 points) and subsequent more invasive re-operations (-14 points) was comparable to the degree of deterioration caused by M as primary breast surgery (-5 + (-14) = -19 points).

As breast cancer treatment oftentimes includes (neo)adjuvant therapies besides surgery, detailed understanding of the associated impact on body image is highly relevant for adequate shared decision-making and postoperative patient care. The present study results indicate that body image deteriorates very much (after Osoba et al. [55]) in the short term when chemotherapy is applied (at therapy initiation -22 points, *p* ≤ 0.0001), but subsequently experiences a recovery over time (+ 15 points per year, *p* ≤ 0.0001). This specifies former results indicating chemotherapy to have a negative influence on body image [25, 42, 67] presumably caused by chemotherapy-associated side effects such as alopecia, weight gain and menopausal symptoms [25, 68]. A recently published large multicentric prospective study by Battisti et al. observed a 10-point lower EORTC QLQ-BR23 body image score six months after diagnosis in patients with chemotherapy (376/1520, 24.7% received chemotherapy) compared to patients without chemotherapy in a group of 1520 high-risk breast cancer patients at age 70 years and older [67]. Unlike the present results, this difference did not reach statistical significance, which might be attributable to the unequal group size (with vs. without chemotherapy) resulting in limited power of the applied linear mixed model [67]. In addition, their results were based on data from older women, who, as observed in the present analysis, seem to report better body image scores compared to younger patients (20 years older associated with 5-point higher body image score). This assumption is supported by Hopwood et al., who observed a negative influence of chemotherapy especially on younger patients’ (< 50 years) body image, mainly caused by alopecia-associated distress in a study population of 2208 breast cancer patients between 26 and 87 years [42]. A linear mixed regression model was applied in the present analysis to capture the complex interactions regarding body image and its influential factors in the best possible way. However, the limitations of a statistical model to fully account for complex reality must be appreciated. Although the estimated weekly change in body image score caused by chemotherapy implies an improvement in body image during chemotherapy treatment, it is more likely to start recovering after chemotherapy is completed and acute side effects have vanished.

In contrast to clinical expectations and former study results [24, 38] radiotherapy was associated with an increase in body image scores at treatment initiation (+ 5 points, *p*≈0.0038), which according to Osoba et al. [55] could be classified as a clinically relevant small improvement. An overall relief perceived by patients after undergoing surgery as a potentially frightening first step of cancer treatment is likely to have caused the improvement in reported body image scores at the beginning of radiotherapy. Presumably, it is after treatment is completed that the patients’ focus shifts from concern over the life-threatening disease towards everyday life and physical appearance leading to adjustments regarding subjective quality of life evaluation. In the present work the effect of radiotherapy over the course of time did not reach statistical significance but indicated a trend towards body image deterioration by about 6 points per year (*p*≈0.1009). Former study results by Rosenberg et al. who investigated body image in 419 young (≤ 40 years) patients within the first six months of breast cancer diagnosis using a multivariable analysis indicated a statistically significant (*p* = 0.01) negative impact of adjuvant radiotherapy on body image [24]. A cross sectional study by Albornoz et al. suggested radiotherapy to be associated with a significantly lower satisfaction with breast and sexual well-being of clinical relevance [38]. The latter study was performed at three centers in USA and Canada to compare body image after implant-based reconstruction without (*n* = 414) and with adjuvant radiotherapy (*n* = 219) using the BREAST-Q questionnaire on average three to four years after breast cancer therapy [38]. In contrast to these two studies the present work analyzed the impact of radiotherapy on body image over time and offers new and more specific insights on its increasing negative effect on body image in the long term [50, 51]. Clinical considerations support an increasingly harmful influence of radiotherapy on body image in the long term for its association with adverse reactions of radiated breast tissue and skin [69].

The present analysis did not find an association between endocrine therapy and body image, which confirms previous study results [42, 70]. It must be mentioned that the EORTC QLQ-BR23 does not encompass items specific for endocrine therapy and its adverse effects and therefore shouldn’t be considered ideal to detect the aforementioned association.

## Conclusions

The present analysis further clarified the impact of different therapeutic strategies for breast cancer on patients’ body image over the postoperative time course. It met the need for knowledge derived from a high-quality prospective study with a rigorous statistical analysis controlling for already known covariables. For the explorative character of this analysis and its uneven size of study groups future studies are needed to confirm the presented findings to be able to inform patients evidence-based as part of preoperative shared decision-making. The following preliminary conclusions are drawn: If feasible, BCS should be offered to patients to minimize damage to their body image [50, 51]. When mastectomy is indicated, immediate breast reconstruction can be offered to mitigate short-term postoperative disruption of body image [50, 51]. However, long-term outcomes after MIBR possibly present a tendency towards deterioration over time [50, 51], whereas after M there seems to be a trend towards recovery. Moreover, attention must be paid to the negative impact of chemotherapy and radiotherapy on body image as they are commonly employed in breast cancer treatment. Chemotherapy seems to harm body image particularly in the short term, while adjuvant radiotherapy is likely to have an additional negative impact on body image in the long term [50, 51] being mandatory after BCS in the treatment of invasive breast cancer. These new insights into body image outcomes after different types of breast cancer treatment will help sensitize caregivers for potential short-term and long-term problems and therefore emphasize the need for discussing postoperative quality of life and offering interventions when needed.

## Supplementary Information

**Additional file 1.** Tumor characteristics, description: table shows tumor characteristics in study population comparing the analyzed surgical groups. 

**Additional file 2.** Response rate for EORTC QLQ-BR23, description: table shows response rate for EORTC QLQ-BR23 questionnaires in study population comparing analyzed surgical groups.

**Additional file 3.** Precision of model, description: table shows values that indicate the precision of the applied linear mixed regression model.

## Data Availability

The datasets analyzed during the presented study are available from the corresponding author on reasonable request.

## Abbreviations

AIC: Akaike-Information-Criterion
ALND: Axillary lymph node dissection
BCS: Breast-conserving surgery
BCT: Breast-conserving therapy
BI: Body image
CI: Confidence interval
DCIS: Ductal carcinoma in situ
DF: Degree of freedom
EORTC: European Organization for Research and Treatment of Cancer
HER2: Human Epidermal Growth Factor Receptor 2
ICHOM: International Consortium for Health Outcomes Measurement
ILC: Invasive lobular carcinoma
M: Mastectomy alone
MIBR: Mastectomy with immediate breast reconstruction
NST: Invasive carcinoma of no special type
PRO: Patient-reported outcomes
QoL: Quality of life
SD: Standard deviation
SE: Standard error
SLNB: Sentinel lymph node biopsy
